# The Natural History of Stingray Injuries

**DOI:** 10.1017/S1049023X22000565

**Published:** 2022-06

**Authors:** Robert J. Katzer, Carl Schultz, Kevin Pham, Micaela A. Sotelo

**Affiliations:** University of California, Irvine, Department of Emergency Medicine, Orange, California, USA

**Keywords:** envenomation, marine, stingray, toxicology

## Abstract

**Introduction:**

Stingray envenomation is a marine injury suffered by ocean goers throughout the world. No prospective studies exist on the various outcomes associated with these injuries.

**Study Objective:**

The aim of this study was to perform a prospective, observational study of human stingray injuries to determine the natural history, acute and subacute complications, prevalence of medical evaluation, and categories of medical treatment.

**Methods:**

This study prospectively studied a population of subjects who were injured by stingrays at Seal Beach, California (USA) from July 2012 through September 2016 and did not immediately seek emergency department evaluation. Subjects described their initial injury and provided information on their symptoms, medical evaluations, and medical treatment for the injury at one week and one month after the injury. This information was reported as descriptive statistics.

**Results:**

A total of 393 participants were enrolled in the study; 313 (80%) of those completed the one-week follow-up interview and 279 (71%) participants completed both the one-week and one-month follow-up interviews. Overall, 234 (75%) injuries occurred to the foot. One hundred sixty-three (52%) patients had complete resolution of their pain within one week and 261 (94%) had either complete resolution or improvement of pain by one month. Sixty-eight (22%) subjects reported being evaluated by a physician and a total of 49 (17%) subjects reported antibiotic treatment for their wound. None of the subjects required parenteral antibiotics or hospital admission.

**Conclusion:**

The majority of stingray victims recover from stingray injury without requiring antibiotics. A subset of subjects will have on-going wound pain after one month. The need for parenteral antibiotics or hospital admission is rare.

## Introduction

Stingrays inhabit coastal tropical and subtropical waters throughout the world. They are common and all share a mutual method of defense: a venomous barb on their tails that can be flexed in any direction to impale an intruder.^1^ These are not infrequently utilized to inflict pain and injury to humans in the water along the beach. Typically, stingray envenomation occurs when a beachgoer inadvertently steps on or next to a stingray in shallow water. Shuffling through the sand with the intention to churn up enough sand to scare off any stingrays before provocation is the typical recommendation to prevent envenomation.^1^

The existing literature on these injuries reflects mostly populations of patients who have presented to an emergency department after injury or called poison control.^2–4^ A retrospective meta-analysis attempted to look at the epidemiology of stingray injuries, but it did not include any prospective studies.^5^ Another large-scale study looked retrospectively at patients who presented to the emergency department with complaint of stingray injury.^1^ It found that of the patients that presented to the emergency department shortly after stingray injury, 88% had their pain relieved with hot water, which is described as non-scalding with an upper temperature of 45^o^C (113^o^F) for 30 to 90 minutes, as a result of deactivation of the venom.^1^ Some also received a single dose of analgesic medication, but none required a second dose. Many of the patients who were discharged without prophylactic antibiotics returned to the emergency department with a wound infection. That study, however, suffered from a significant selection bias and, by design, was unable to quantify the portion of the patient population stung by stingrays that experienced complications and subsequently developed cellulitis, sought medical care elsewhere, or required other interventions. Several other case studies described other injuries such as vascular injury, associated arrhythmias, and soft tissue infection.^6–8^ None of these studies followed patients prospectively from the time of the injury. One paper from the 1950s reported that only 20% of stingray-envenomated patients eventually seek medical care.^9^ Several forms of treatment such as hot water treatment and antibiotics have been proposed and utilized to alleviate pain and prevent infection in this patient population.^1–3,5,9,10^ As these studies are all based on outcomes in very selective patient populations, the results are heavily biased.

No number of retrospective studies will resolve these problems. However, a potential solution to these challenges is possible. Large populations of stingrays often reside in high densities at particular beaches. Seal Beach, California (USA) has one of the highest annual numbers of reported stingray injuries on the California coast, to the order of 500 or more annual reported stings.^11–15^ The lifeguards there are very accustomed to addressing these patients and taking detailed records of the injuries. A prospective evaluation of this human population followed from the time of stingray envenomation to complete recovery could yield answers by finally providing both the numerator and the denominator for many of these measurements. This study proposed to achieve just that.

Currently, no prospective data exist describing the natural progression and resolution of human stingray envenomation, the associated disease complications, the need for medical intervention, and efficacy of treatment, if any. Without such prospective natural history information, obtaining accurate estimates for any of these disease parameters is difficult. However, a study that follows a patient population from the moment of envenomation through the entire time course of their disease may provide accurate outcome estimates that will allow physicians to better evaluate and treat these patients.

The purpose of this study was to prospectively collect data on individuals envenomated by stingrays to better understand the natural history of the disease. These data focused on the course of the injury, risk of complications (both acute and subacute) such as retained foreign bodies and infection, the utilization of intervention (radiography, surgery, hospitalization), and efficacy of treatment modalities, including hot water and the need for antibiotics. The goal was to obtain a more accurate estimate of the entire injury course by identifying patients immediately after injury instead of waiting for some of them to seek medical care. This allowed a more accurate analysis of stingray injuries by obtaining both the denominator of overall exposure as well as the frequency and duration of particular symptoms, medical evaluations, and treatments.

## Methods

The study was a prospective observational study of subjects who suffered a stingray injury while in the water at Seal Beach, California from July 2012 through September 2016.

The study was approved by the University of California, Irvine Institutional Review Board (Orange, California USA; HS# 2011-8620). The study enrolled a consecutive sample of participants who were identified by a lifeguard at Seal Beach, California (located at the latitude and longitude of 33.738, -118.107) as having a stingray injury. The study aimed to enroll between 300 and 450 study subjects based on the previously reported incidence of significant injury after stingray envenomation.^4^ The study’s geographic area was most commonly populated by the round stingray, *Urobatis halleri*.^11^ The study enrolled consecutive subjects who were identified by a lifeguard at Seal Beach, California as having a stingray injury. The beach area is open for the public and staffed with city lifeguards starting at 08:00am all year. Beach lifeguard staffing continues until between 5:00pm and 10:00pm each day, depending on the season and if it is weekday versus weekend. Lifeguards are required to be trained in cardiopulmonary resuscitation/CPR and first aid. As part of standard procedure, beachgoers with stingray injuries were brought to an outdoor assessment area where their wound was evaluated and immersed in non-scalding hot water to deactivate the stingray venom, as available from the standard local hot water fixture, until pain was controlled, which was typically between 30 and 60 minutes in the agency’s experience. If there was any readily removable stinger barb, lifeguards would provide the first aid wound care of removing it. If a beachgoer had on-going bleeding, uncontrolled pain, or otherwise requested ambulance transport to an emergency department, the lifeguards would request ambulance transport. While receiving hot water treatment, beachgoers were shown a flyer about the study. If the beachgoer was interested in enrolling, the lifeguard called a designated phone number where a research assistant would consent and enroll the participant over the phone. Research assistants consisted of a group of undergraduate students who worked shifts in an academic medical center emergency department, enrolling patients in on-going department research projects. They were on duty daily between 8:00am and 12:00am/midnight. If the lifeguards were unable to get in touch with the research assistants, they would call the principal investigator directly to consent and enroll the participant. During enrollment, study participants provided research personnel with their name, date of birth, as well as primary and secondary telephone contact numbers. They also provided personnel with the most convenient hours of the day to reach them via telephone. In the event that the participant was a minor, they gave their verbal assent and a parent or guardian was contacted for verbal consent.

Study participants were contacted by research personnel by telephone both one week and one month after the date of the injury. For both follow-up phone calls, personnel conducted a scripted interview which included the questions in Table 1 and Table 2. The responses were recorded on a standardized study participant paper response sheet with defined data elements. If the participant did not answer the telephone, a message was left with the research call back number. If contact did not occur on initial attempted contact, one additional call was attempted two hours later that day. If the participant did not answer the second time, a final attempt was made the next day. If all three attempts were unsuccessful and the patient did not return the call after voicemail, that patient was considered lost to follow-up. To collect the lifeguard assessment and treatment data, study personnel periodically visited the lifeguard office and recorded information that the lifeguards documented in the treatment record. The data points collected were entered into a spreadsheet using Microsoft Excel Version 16.55 (Microsoft Corporation; Redmond, Washington USA). Data collected from follow-up phone calls were also compiled into the spreadsheet corresponding to each participant. Participants that could not be contacted for one-week follow-up were excluded. Data from the lifeguard treatment record were analyzed only for cases where participants provided at least one-week follow-up data. Participants lost to follow-up during the one-month interview were noted, but their one-week follow-up and lifeguard treatment records were analyzed as part of the group. If discrete data from the lifeguard treatment record were not present for a patient, this was reflected in the adjusted N value on the results associated with that data element.

**Table 1.** Injury Assessment Questions

Circumstances of the Injury	Symptoms
What were you doing?	Pain?
Approximately how deep was the water?	Rash?
Where on your body were you stung?	Difficulty breathing?
Did you appear to be stung in one location or multiple locations?	Palpitations or heart pounding?
Is this your first stingray injury?	Abdominal pain?
	Vomiting?
	Weakness?
	Swelling or tenderness to the lymph node areas of the groin, neck, or armpit?
	Fainting?
	Bleeding? If so, how long and how was it stopped?
	Low blood pressure?
	Anything else?

**Table 2.** Assessment and Treatment Questions

Medical Assessment	Treatment
Did you see a doctor for the injury?	Washing of the wound by a medical professional?
If so, was it your primary doctor, urgent care, the ER, or another type of doctor?	Removal of foreign body?
What evaluation did the doctor perform?	IV fluids?
Physical exam?	Pain Medication: IV, oral, prescription, narcotic?
Blood tests?	Hot water immersion?
X-rays?	Injected local pain medication such as lidocaine?
Tissue sample for biopsy?	Vinegar?
Ultrasound?	Stitches?
MRI, CT scan, or other advanced imaging?	Antibiotics, if so and if you remember what was the name and how many days did you take them for?
Did you miss work as a result of the injury, and if so, for how long?	Admission to the hospital?
Repeat doctor visit for a wound evaluation?	Surgery?
Any other assessments?	Hyperbaric oxygen treatment?
	Stitches?
	Other topical treatment?
	Any other treatment?

Abbreviations: ER, emergency room; MRI, magnetic resonance imaging; CT, computerized tomography; IV, intravenous.

### Outcome Measures

The study’s primary outcome was description of the symptoms and their duration, medical assessment, and treatment subjects underwent after suffering a stingray injury. These included, but were not limited to, the location of the sting, severity of pain, bleeding, medication utilized, physician visits, diagnostic tests, hospital admissions, and missed work. The study measured these out to one month from the injury using descriptive statistics.

### Statistics

Frequencies are presented as N (%). In univariable analysis, chi-square test was used to examine the association of different symptoms and management. In multivariable analysis, the ordinal logistic regression analysis was used to assess the association of pain at one week with antibiotic use, rash, and swelling at that time. A P value less than .05 was considered statistically significant. Data were analyzed by using STATA 14 (Stat Statistical Software: Release 14; StataCorp 2015: College Station, Texas USA).

## Results

A total of 393 participants were enrolled from July 2, 2012 through September 18, 2016; 279 (71%) of these were male and 93 (24.8%) of these were female. Twenty-one (5%) did not report their gender. Overall, 313 (80%) of those completed the one-week follow-up interview and 279 (71%) of those completed both the one-week and one-month follow-up interviews. The most common location of sting was the foot (232; 75%), followed by the toe (47; 15%), the ankle (25; 8%), and other (7; 2%). “Other” was defined as either a location aside from the above three or as multiple locations. Two participants did not report the location of the injury.

One week after the injury, 163 (52.1%) of the 313 interviewed patients no longer had pain associated with the wound. By one month after the injury, of the 279 patients who were interviewed, 187 (67%) patients noted that their pain had completely resolved and 74 (26.5%) patients noted their pain had improved. Three (1.1%) patients did not address their one-month pain state and 15 (5.4%) patients reported on-going, unchanged pain from the wound.

Sixty-eight (22%) participants visited a physician for their injury. Four participants did not specify whether or not they visited a physician. Of those that visited a physician, 53 (78%) visited urgent care or their primary care physician. Nine (13%) went to an emergency department. Evaluation by a physician was associated with the presence of pain at one week (P = .02), however not the initial presence of pain (P = .177). Three (0.8%) patients obtained x-rays. One (0.3%) patient obtained an MRI [magnetic resonance imaging]. Two patients reported having a foreign body removed from the wound.

Fifty-four (17%) participants reported antibiotic treatment within one month of the initial injury. The different antibiotic categories are found in Table 3. Cephalexin (ten patients) and Doxycycline (eight patients) were the most commonly prescribed antibiotics. In ordered logistic regression analysis, as displayed in Table 4, the presence of pain at one month was associated with the presence of pain at one week (P = .004) as well as with the use of antibiotics (P = .000). The presence of pain at one month was not associated with the presence of rash (P = .501) or swelling (P = .462). An analysis of variance of initial pain severity by pain at one month did not demonstrate a relationship (P = .66). Non-steroidal anti-inflammatory drugs were the most common agent used to treat pain. The prevalence of each pain control agent type is listed in Table 5. None of the patients were admitted to the hospital. Of the enrolled patients, none of them were one-month transported from the scene to the hospital.

**Table 3.** Antibiotic Treatment

Type of Antibiotic Given	Number of Patients	Number of Patients, Including Those on More Than One Antibiotic
No Antibiotic	259 (83%)	N/A
Penicillin	6 (2%)	6 (2%)
Quinolone	3 (1%)	4 (1%)
Cephalosporin	10 (3%)	12 (4%)
Bactrim	2 (<1%)	5 (2%)
Doxycycline	3 (1%)	8 (3%)
Other	25 (8%)	25 (8%)
More than One Antibiotic	5 (2%)	N/A
Total	313	60 (19%)

**Table 4.** Ordinal Logistic Regression Analysis of Predictors of Pain Severity after One Month

Variables	Coefficient	Standard Error	P Value
Pain at Week 1	0.70	0.275	.010
Antibiotic Use	1.63	0.332	<.001
Rash	−0.54	0.444	.227
Swelling	−0.46	0.505	.362

Note: Number of observations: 274; Pseudo R2 = 0.08; Dependent variable: pain after one month (same, better, resolved).

**Table 5.** Pain Management Methods

Type of Pain Management	Number of Patients	Percentage of Total Patients
Acetaminophen	7	2.2%
Opioid	5	1.6%
Non-Steroidal Anti-Inflammatory Drug	71	22.7%
Oral Ethanol	2	0.6%
Other	12	3.8%
No Pain Management	186	59.4%
Did Not Report Pain Management	33	10.5%

Note: Three patients used more than one type of pain management, resulting in a total percentage greater than 100%.

## Discussion

This study provided prospective data on the symptoms and their duration, common treatment, and complications of stingray injuries. This study demonstrated that the majority of patients do not require follow-up with a physician, as 245 (78%) of study subjects did not seek further care. These findings support a wait-and-see approach to initial management of stingray injuries.

Previous studies have examined patients who have sought care in the emergency department for a stingray injury and documented their need for pain control as well as their rate of return with signs and symptoms of infection.^2,3^ However, these investigations suffered from significant selection bias by only evaluating patients whose signs or symptoms were concerning enough that they sought emergency care. These studies could not provide more detailed information on the frequency of such complications in the general population of envenomated victims. No other study has examined a population of stingray injury victims in a prospective manner. Although this study undoubtedly failed to capture all stingray victims from Seal Beach during the study period, it did collect data on the population of patients who did not seek care in an emergency department, which is a population that had not previously been evaluated.

## Limitations

This study has several different limitations. The study subjects only included victims of stingray injury that were cared for by a Seal Beach lifeguard and who volunteered to participate in the study. While it is possible a rare individual stung by a stingray could walk off the beach and not be seen by the lifeguards, and thus not be captured for the study, this is highly unlikely. The large distance of sand between the beach and the parking lot makes it extremely difficult to walk to your car while experiencing the pain of an envenomation. As such, the vast majority of people stung by stingrays make contact with a lifeguard. None of the stingray victims who were transported via ambulance were included in the study. It is possible that only those stingray victims with less severe symptoms elected to participate in the study. A further limitation is that all patients enrolled in the study received the standard of care wound treatment by the lifeguards with hot water immersion for a period of approximately one hour.^1^ As a result, this study cannot draw any conclusions about the natural course of stingray injuries that do not receive hot water treatment shortly after the injury occurred. Furthermore, although the study collected information on diagnostic evaluation and treatment that the subjects received, it cannot provide any conclusions on the efficacy of these interventions due to the observational nature of this study. Finally, 80 (20%) of the study subjects were lost to follow-up at one week and 114 (29%) were lost to follow-up at one month. The study was unable to assess how the symptoms and treatment of those subjects would have affected the study’s overall data.

## Conclusion

This study concluded that the majority of stingray victims will recover from their injury without the use of antibiotics or other medical intervention. This investigation did suggest that a subset of subjects exists who will experience on-going pain at the wound site for at least one month. It also indicated that the need for additional diagnostic testing, parenteral antibiotics, or hospital admission is rare. As many different stingray species have similar venom, some of the conclusions of this study involving pain may apply to other species throughout the world.^1^ The data regarding the incidence of infectious symptoms and type of antibiotic utilized may be dependent to some extent on the bacterial species and concentration within the local waters of the injury. The baseline data provided by this study should allow investigators in future studies to look at treatment for pain and consider the limitations of infection prophylaxis.
